# Strict or Graduated Punishment? Effect of Punishment Strictness on the Evolution of Cooperation in Continuous Public Goods Games

**DOI:** 10.1371/journal.pone.0059894

**Published:** 2013-03-28

**Authors:** Hajime Shimao, Mayuko Nakamaru

**Affiliations:** Department of Value and Decision Science, Tokyo Institute of Technology, Meguro-ku, Tokyo, Japan; University of Maribor, Slovenia

## Abstract

Whether costly punishment encourages cooperation is one of the principal questions in studies on the evolution of cooperation and social sciences. In society, punishment helps deter people from flouting rules in institutions. Specifically, graduated punishment is a design principle for long-enduring common-pool resource institutions. In this study, we investigate whether graduated punishment can promote a higher cooperation level when each individual plays the public goods game and has the opportunity to punish others whose cooperation levels fall below the punisher’s threshold. We then examine how spatial structure affects evolutionary dynamics when each individual dies inversely proportional to the game score resulting from the social interaction and another player is randomly chosen from the population to produce offspring to fill the empty site created after a player’s death. Our evolutionary simulation outcomes demonstrate that stricter punishment promotes increased cooperation more than graduated punishment in a spatially structured population, whereas graduated punishment increases cooperation more than strict punishment when players interact with randomly chosen opponents from the population. The mathematical analysis also supports the results.

## Introduction

Cooperative behavior is commonly observed in animal populations and between human beings. This behavior in wild animals can be largely explained by kin selection [1], [2]. For human beings, cooperative behavior is not only directed toward kin but also frequently found toward non-kin. Therefore, from an evolutionary viewpoint, understanding cooperative behavior has been problematic because cooperators are often inferior in fitness to defectors who do not pay costs to cooperate and benefit from the cooperators’ actions. The Prisoner’s Dilemma game describes this situation. Two players, who cannot bargain with each other, can either cooperate or defect. A cooperative player gives benefit *b* to the other player and pays the cost of cooperation, −*c* (*b*>*c* >0). A player who defects never gives anything to the other player. If both cooperate, they both score *b* − *c*. If one cooperates and the other defects, the cooperator’s score is −*c* and the other’s is *b*. If both choose defection, both score 0. In the classic Prisoner’s Dilemma game, both players choose to defect and receive a score of 0 although the score (*b* − *c*) for both players would be higher if they both cooperate. Therefore, the Prisoner’s Dilemma game describes the failure of mutual cooperation between two players.

The conditions or mechanisms that influence people’s cooperation have been studied using the Prisoner’s Dilemma game, public goods game, and other approaches [3]. The public goods game describes social dilemma during collective action. Each player invests his/her resources in public goods. The pool of investments from all players is multiplied by a benefit factor and divided equally among all players. The payoff function suggests that when all players invest in public goods, the payoff is higher than when they do not invest at all. However, if one player changes from cooperation to defection, the player gains a higher payoff than what she/he would have gain under cooperation. Thus, all players would choose defection. This is known as a social dilemma. These investigations have identified a variety of factors that affect the evolution of cooperation, such as direct reciprocity [4], indirect reciprocity [5], group selection [6], [7], and spatial structure [8]–[13]. Among these mechanisms, costly punishment has attracted the most attention [14]–[22]. This idea introduces another stage, a punishment stage, into the prisoner’s dilemma game or the public goods game. In the punishment stage, a punisher, who can be either a cooperator or defector would attack an opponent as retribution if the opponent defected in the first stage. In the early model of Axelrod [15], a punisher had to be a cooperator, but a paradoxical strategy that allows defectors to punish is now considered [23]–[26]. Obviously, defections are less rewarded if the opponent is a punisher.

Evolutionary game studies have demonstrated that cooperation can evolve or be maintained if punishment evolves [16], [27]. However, we must ask whether punishing behavior itself evolves because while a punisher must pay a cost, a non-punisher does not, and thus often outperforms the punisher. This problem is called the second-order dilemma and mechanisms have been suggested to solve it. Fehr and Gächter argued that people often punish defectors whom they will never meet again; this is altruistic or cooperative behavior [28]. They also stated that the primary reason for punishment in this case stems from a negative reaction toward defectors [29]. If so, punishment might be, as much as cooperation, a product of evolution and natural selection. We then must ask why and how these behaviors evolve.

One solution for the evolution of costly punishment is to adopt a setting that allows punishment to have fitness advantages [24], [30], [31]. As Nakamaru and Iwasa demonstrated, if spiteful behavior which is defined as a behavior that decreases an opponent’s score by reducing one’s own score can have relatively high fitness, it may evolve with an updating rule called the score-dependent viability model or simply the viability model [23], [30]. In this model, an individual’s score is inversely proportional to the probability of that individual’s death. When the individual dies, a randomly chosen player procreates and fills the empty site. With this action, spiteful behavior results in some advantage in relative fitness despite its cost because, if a spiteful individual can decrease the opponent’s score, the death probability is high enough to empty the opponent’s site, and then the individual has the chance to colonize the empty site. Spiteful behavior works effectively in a spatially structured population such as a lattice if a focal individual interacts with the neighbors and has the chance to colonize an empty site, which is created after the neighbor dies. As a mechanism, punishment can be considered spiteful, as it reduces both the opponents’ and one’s own scores. Therefore, punishment can evolve in the same manner as spiteful behavior. As a result, punishment can increase cooperation in the viability model if an individual behaves as both a punisher and cooperator.

The other widely studied concept is that of spatial structure. In a spatially structured population, players are distributed within a structure, such as a lattice, or other networks or subpopulations, and they interact locally only with their neighbors. Brandt et al. assumed that each player plays the public goods game with two other players in a population with a hexagonal lattice structure and showed that cooperation and punishment can evolve together [32]. They also showed that cooperation can increase without punishment if a spatial structure exists, but the existence of punishment more strongly increases cooperation. Other studies have shown that a spatially structured population can resolve a second-order dilemma [33], [34].

Although numerous models of punishment have been developed, most assume discrete or binary strategies for both cooperation (cooperator or defector) and punishment (punisher and non-punisher). Brandt et al., for example, assumed four strategies: cooperate and punish, defect and punish, cooperate but do not punish, and defect but do not punish [32]. These simple settings are convenient for mathematical analysis and are thus helpful for the discussion of basic mechanisms. However, in reality, the cooperation level or the amount of contribution to the public goods in the public goods game is continuous [35], [36]. The cooperation level in certain collective actions is continuous, and there may be several discrete cooperation levels, e.g., levels 1, 2, …,10. We may consider the example of house cleaning by several housemates. Some housemates help clean the house completely, some do so almost completely, some do a bit, whereas others do not help at all. Thus, a continuous cooperation level can encompass a situation in which there are more than two choices. The punishment level is also continuous depending on the level of continuous cooperation. If we adopt these realistic assumptions, we can investigate the punishment level in response to the opponent who contributes a certain level of cooperation to the public goods.

Nakamaru and Dieckmann investigated the effect of strictness of punishment on the evolution of the continuous cooperation level [37]. Strict punishment means a policy of “zero tolerance”: a punisher severely punishes an opponent whose cooperation level is below the threshold and weakly punishes an opponent whose cooperation level is above the threshold. Nakamaru and Dieckmann concluded that, in a population with a lattice structure, the rule “the stricter, the better” had to be applied to punishment if cooperation were to evolve. They also mathematically proved that if each individual interacts with a randomly chosen individual from the population, then neither cooperation nor punishment can evolve. Gao et al. also studied the evolution of the continuous cooperation level and punishment in a spatially structured population [38] and implicitly assumed strict punishment. They discussed the effect of social tolerance corresponding to the threshold of punishment [37] on the evolution of cooperation and punishment.

If punishment is graduated, a punisher gradually changes the severity, adjusting to the cooperation level. This principle is followed by the criminal laws of most western countries [39]. Other examples include the following. Ostrom found that graduated punishment is one of seven design principles for long-enduring common-pool resource institutions and graduated punishments for violators are likely to be assessed depending on the seriousness and context of the violation [40]. Cox found that graduated punishments progress on the basis of either the severity or repetition of violations to deter participants from excessive violations of community rules [41]. In many legal systems, repeat offenders are punished more severely than first-time offenders, and theoretical studies of criminal sanctions have shown that the erroneous conviction of innocent offenders and learning contribute toward making this sanction system optimal [42]–[44].

Therefore, in this study, we investigate whether graduated punishment depending on the cooperation level increases cooperation in the continuous public goods game. We also investigate how spatial structure affects the results; this outcome depends on an updating rule that prescribes how the game score affects fitness and generational changes. Updating rules can dramatically change evolutionary dynamics. For example, Nakamaru and Iwasa [23], [30] investigated whether the conditions for the evolution of cooperation differ between two different updating rules: the score-dependent viability model and the score-dependent fertility model, which is the same as “the death-birth” model [45]. They found that punishment and cooperation can evolve in both the completely mixed population and the spatially structured population when using the viability model, whereas the coevolution of cooperation and punishment is impossible without the spatial structure when using the fertility model. Therefore, in contrast to Nakamaru and Dieckmann [37], we expect that the viability model can foster the coevolution of cooperation and punishment even in a completely mixed population.

### Models

We assumed a population consisting of *N* × *N* individuals. Each individual *i* has four adaptive traits: propensity for altruism (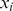), severity of punishment (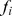), threshold of punishment or tolerance level (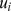), and strictness of punishment (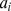). Higher values of *x* and *f* represent greater cooperation and more severe punishment, respectively. The values of *u* and *a* determine the punishment function. The value of *u* is the threshold of the cooperation level, below which the focal individual punishes the opponent, and the value of *a* determines the level of punishment in response to the opponent’s cooperation level. The former three parameters are continuous and limited to the range 0–1. Strictness is also continuous but ranges from 0 to infinity. During each turn, an individual *i* is randomly chosen from the population and plays the two-stage game in each group of four members, which includes individual *i*.

The two-stage game consists of the cooperation and punishment stage. In the cooperation stage, the public goods game is played; each member contributes his/her resource to the public goods. The amount of contributions is determined by their propensity for altruism *x*. The pool of contributions from all the members is multiplied by *r*, defined as the efficiency of public goods, and equally divided among the four members so that the score for an individual *i* with a cooperation level 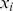 after the cooperation stage ends is
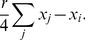
(1)


In the punishment stage, the four members punish each other according to the others’ cooperation levels and pay the cost of punishment. The cost that an individual *i* pays to punish a member *j* whose cooperation level is *x_j_* is defined as
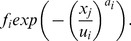
(2)


**Figure 1:**
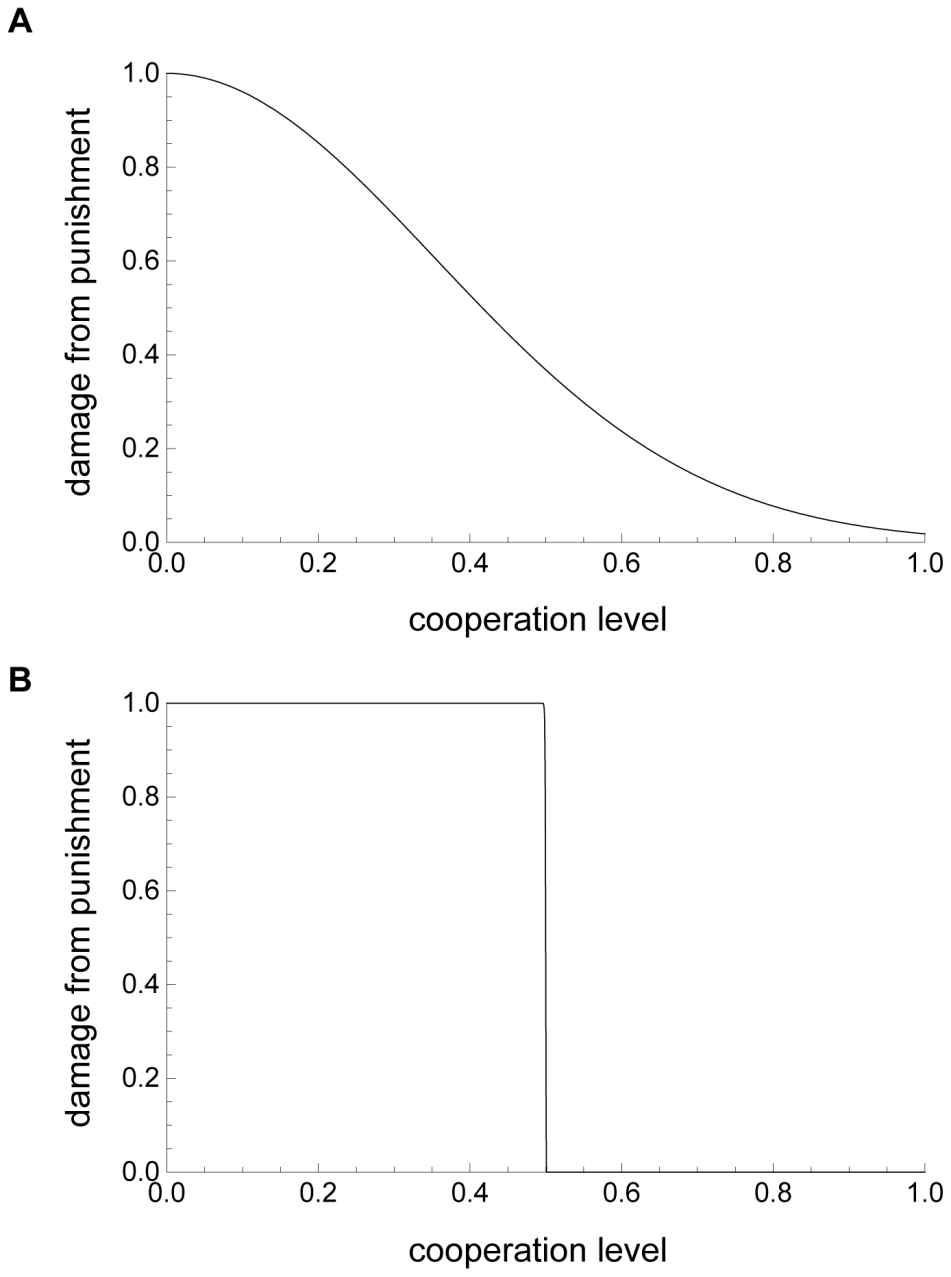
Punishment severity. (a) and (b) show the relationship between the cooperation level of the opponent (horizontal axis) and the damage from punishment (vertical axis) when punishment is graduated (*a* = 2) and strict (*a = *1,000), respectively. The other parameters are *f = *1.0, *u* = 0.5, and *β* = 1.0.

Figures 1a and 1b present the examples of punishment when *a* = 2 and *a* = 1,000, respectively.

The damage suffered by a punished opponent is
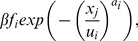
(3)where *β* (the efficiency of punishment) is above 1. These functions represent the relationship between the strength of punishment and the opponents’ cooperation level. If *a* is small, the strength of punishment gradually decreases as the cooperation level increases, whereas it decreases steeply around the threshold *u* if *a* is high.

The total score of an individual *i* (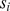) in a game is the sum of (i) the benefit and cost of the public goods game, (ii) the cost of punishing the other three opponents in the group, and (iii) the damage from the other members’ punishment. That is,
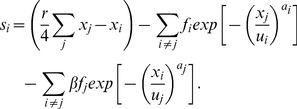
(4)


After the two-stage game, the death and birth events occur. In this study, we used an updating rule called the score-dependent viability model or viability model, in which death probability is inversely proportional to one’s score. Here, the death probability of individual *i* is
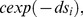
(5)where *c* and *d* are constant and positive and 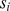 is the individual’s score.

If an individual *i* dies as predicted by his death probability, a new empty site exists. One of the other individuals reproduces and that individual’s offspring immediately fills the empty site. The offspring inherits the four adaptive traits of the parent, and mutation occurs with probability *m* for *x*, *u,* and *f* and *m_a_* for *a*. If a mutation occurs, the offspring’s trait is normally distributed around the parental trait, with standard deviation *σ* for *x*, *u,* and *f* and *σ_a_* for *a*. In all the results presented in this paper, we set these parameters as *m* = 0.01, *m_a_* = 0.1, *σ* = 0.01, *σ_a_* = 1.0, and *N* = 50. At this point, one turn ends. We did not run simulations in a lattice larger than *N* = 50. If a larger lattice was used, we would have expected the simulation outcome to be almost the same as that presented in this paper.

Our assumptions differ from those of Nakamaru and Dieckmann [37]. Unlike Nakamaru and Dieckmann, we did not allow the existence of empty sites in the spatial structure. Sekiguchi and Nakamaru demonstrated that in the score-dependent viability model, the existence of empty sites promotes the evolution of altruistic punishers more than the non-existence of empty sites [46]. Thus, it is more difficult for punishment and cooperation to evolve in our setting than in that of Nakamaru and Dieckmann.

If evolutionary dynamics is interpreted as social learning, the death probability (eq. 5) can be considered the probability of a change in behavior. The individual decides to change the behavior when the behavior’s score is low. After deciding to change the behavior, the individual imitates and exhibits another behavior randomly chosen from the neighbors or the population.

In each generation, *N* × *N* turns are completed; thus, every individual experiences this birth and death process once on average.

We compared two conditions for spatial structure: the spatially structured condition and the random-matching condition. These two conditions differ in whom each individual interacts with and who produces offspring in the empty site created by a death. By comparing the results under these two conditions, we investigated how spatial structure affects the evolution of cooperation and punishment.

First, let us describe the spatially structured condition. We assumed a population consisting of *N* × *N* individuals set in an *N* × *N* square lattice. Under these conditions, each individual plays the public goods game with their nearest neighbors. We assume that all the payoffs for the focal player are totaled for all the games played by the focal player. Let us explain our detailed assumptions. We assume that one group has four members who play the public goods game and decide to punish other members in the same group. In the Moore neighborhood, each individual has eight nearest neighbors. If we assume that four nearest neighbors make one group, each individual belongs to four groups. For example, the focal player is known as X, and X’s eight neighbors are known as A, B, C, D, E, F, G, and H, who are positioned clockwise from the top. X belongs to four groups. The first group comprises A, B, C, and X; the second, C, D, E, and X; the third, E, F, G, and X; and the fourth, G, H, A, and X. Therefore, the total payoff for X is derived from the payoff accumulated in all four groups. After the focal individual dies according to the total score, one of the eight neighbors is randomly chosen to reproduce and fill the empty site with an offspring.

Second, we explain the random-matching condition. One individual and eight opponents are randomly chosen from the entire population during each turn. The selected eight opponents are put into the Moore neighborhood, and the interaction process and the reproduction process are the same as those in the spatially structured condition. After one turn, a new focal individual and eight new opponents are again randomly chosen and the game and reproduction process start anew. This process repeats until the end of the simulations (1,000,000 generations). Thus, each individual plays the public goods game with almost different opponents in each generation.

We made the complicated assumption about the interaction partners to use the same assumption as the spatially structured condition and compare the results of the two conditions.

## Results

The results of the simulations indicated that determining whether punishment should be strict or graduated for the evolution of cooperation depends heavily on how constantly the individuals interact with their neighbors. Note that all the values below are the average of 50 simulation trials.

**Figure 2:**
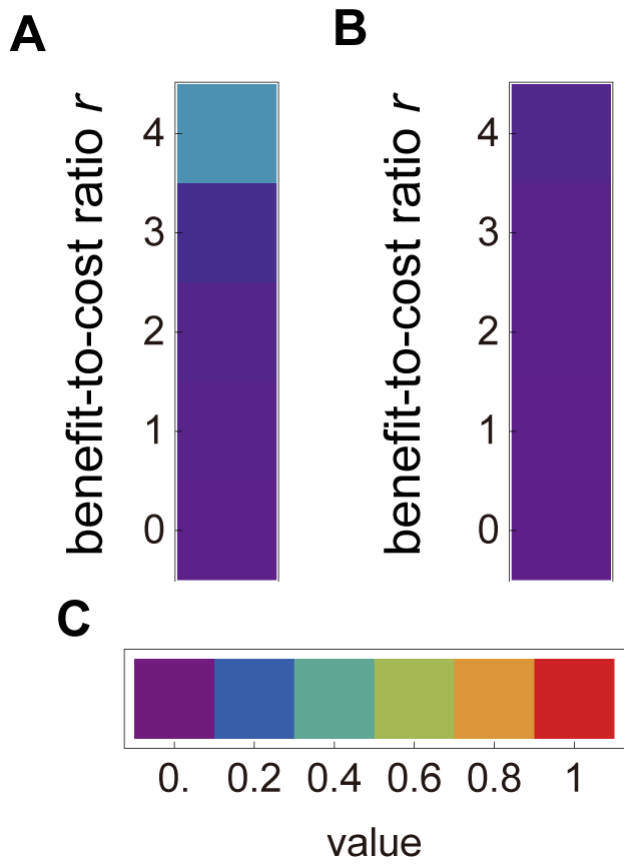
Effectiveness of public goods (*r*) and evolution of cooperation level without punishment. (A) and (B) present the cooperation level after 1,000,000 generations in the spatially structured condition and the random-matching condition, respectively. Other conditions are the same as noted in the main text. Each data block is the average of 50 trials. (C) presents the relationship between a value and color in both (A) and (B).

First, to distinguish the effect of the lattice from that of punishment on the evolution of the cooperation level, we examined whether the lattice structured population can increase the cooperation level without punishment. Fig. 2 depicts the outcomes of the evolutionary simulations and illustrates the cooperation level’s slight increase in *r* = 4, but not in *r* <4 in the spatially structured condition (Fig. 2A). Fig. 2B illustrates the cooperation level’s complete failure to increase in the random-matching condition. These results indicate that the cooperation level does not increase without punishment regardless of the spatial structure.

**Figure 3:**
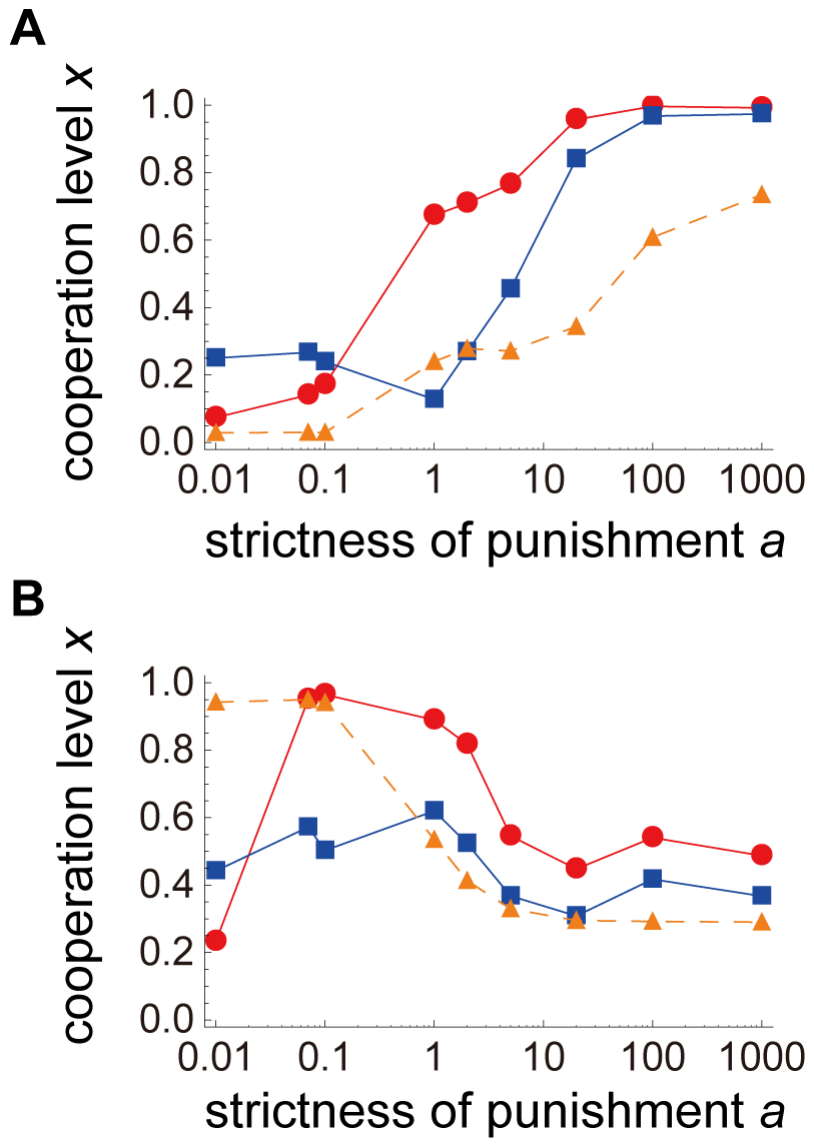
Evolution of cooperation level, punishment severity, and punishment threshold in the spatially structured condition. Values are averages of 50 trials at 1,000,000 generations. The horizontal and vertical axes represent log *a* and the value of each trait, respectively. The cooperation level (*x*) is denoted by a solid red line and points, the punishment severity (*f*) is denoted by an orange dotted line and triangles, and the punishment threshold (*u*) by a solid blue line and squares. The final level of cooperation with no punishment is 0.06272092 in the spatially structured condition and 0.03539616 in the random-matching condition. (A) presents the spatially structured condition and (B) the random-matching condition. The value of *a* used in this figure is 0.01, 0.07, 0.1, 1, 2, 5, 20, 100, and 1,000. When *a* = 0.0, (*x*, *f*, *u*) = (0.0747, 0.0327, 0.3223). The parameters are *r* = 3, *β* = 10.

Next, we examined how spatial condition and strictness of punishment (*a*) affect the evolution of each trait when the strictness of the punishment was fixed for all individuals (Fig. 3). In the initial state, all traits were zero, that is, at first there was no punishment or cooperation. The evolved level of cooperation with punishment was much higher than that without punishment (Fig. 3A) and we concluded that punishment could increase cooperation. Cooperation and punishment evolved to greater levels with strict punishment than with graduated punishment in the spatially structured condition (Fig. 3A). These results were compatible with those of Nakamaru and Dieckmann [37] despite the differences in assumptions and procedures. In contrast, in the random-matching condition, the simulation outcomes demonstrated that cooperation and punishment evolved with graduated punishment (low *a*) rather than severe punishment (Fig. 3B). If *a* is too low (roughly 0.0), where the punishment level is nearly the same regardless of the opponent’s cooperation level, the cooperation level will not increase.

**Figure 4:**
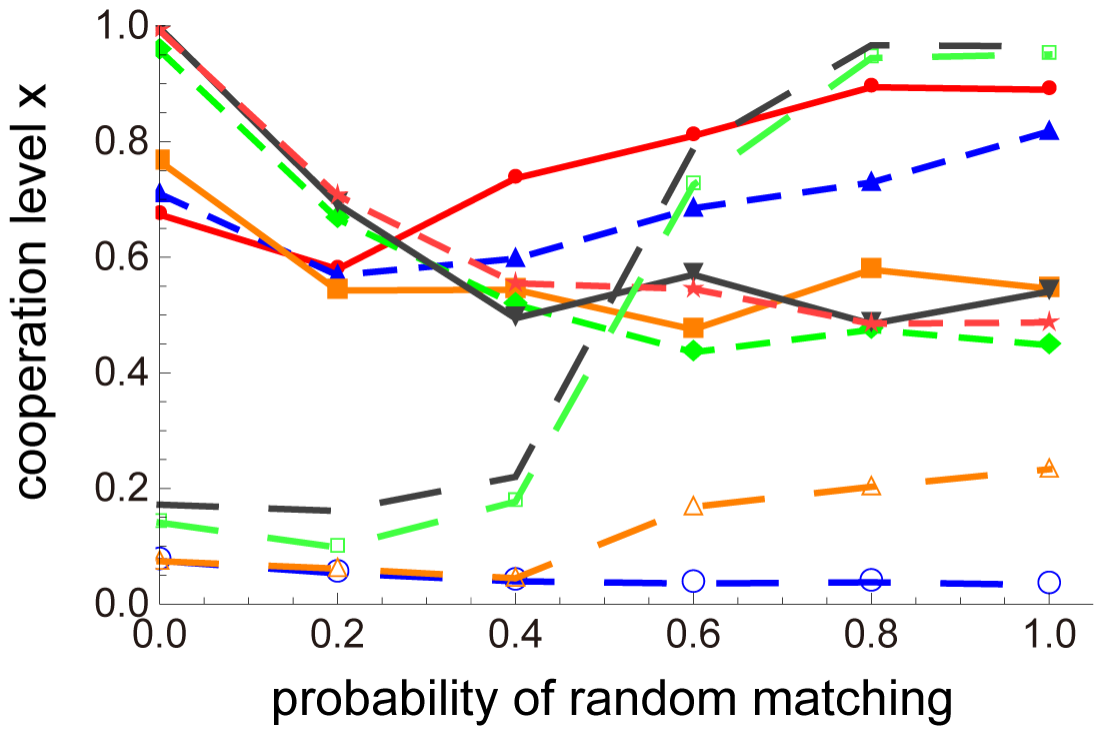
Effect of randomness on evolution of cooperation level when *a* is fixed. The horizontal and vertical axes represent randomness (*p*) and the value of the cooperation level (*x*), respectively, after 1,000,000 generations. The dotted blue line with open points is the average *x* of 50 trials of *a* = 0.0; the orange dotted line with open triangles, *a* = 0.01; the green dotted line with open squares, *a* = 0.07; the black dotted line, *a* = 0.1; the solid red line with points, *a* = 1; the dotted blue line with triangles, *a* = 2; the orange solid line with squares, *a* = 5; the green dotted line with diamonds, *a* = 20; the black solid line with inverted triangles, *a* = 100; and the red dotted line with stars, *a* = 1,000. Other parameters are the same as in Fig. 3.

Fig. 4 depicts the effect of spatial randomness on the evolution of cooperation level given the strictness of the punishment. Neighbors of a focal player and randomly chosen players from the population are selected as opponents of the focal player with a probability of 1 − *p* and *p*, respectively. The spatially structured condition corresponds to *p* = 0 and the random-matching condition corresponds to *p* = 1. When strictness (*a*) was higher, less randomness increased the cooperation level, whereas with increasingly graduated punishment (lower *a*), increasing randomness increased the cooperation level. Specifically, when *a* was roughly 0.1, the difference in *x* between *p* = 0 and *p* = 1 was the largest. Regardless of randomness, the cooperation level did not increase when the punishment level was not dependent on the cooperation level (*a* = 0.0 or 0.01 in Fig. 4).

**Figure 5:**
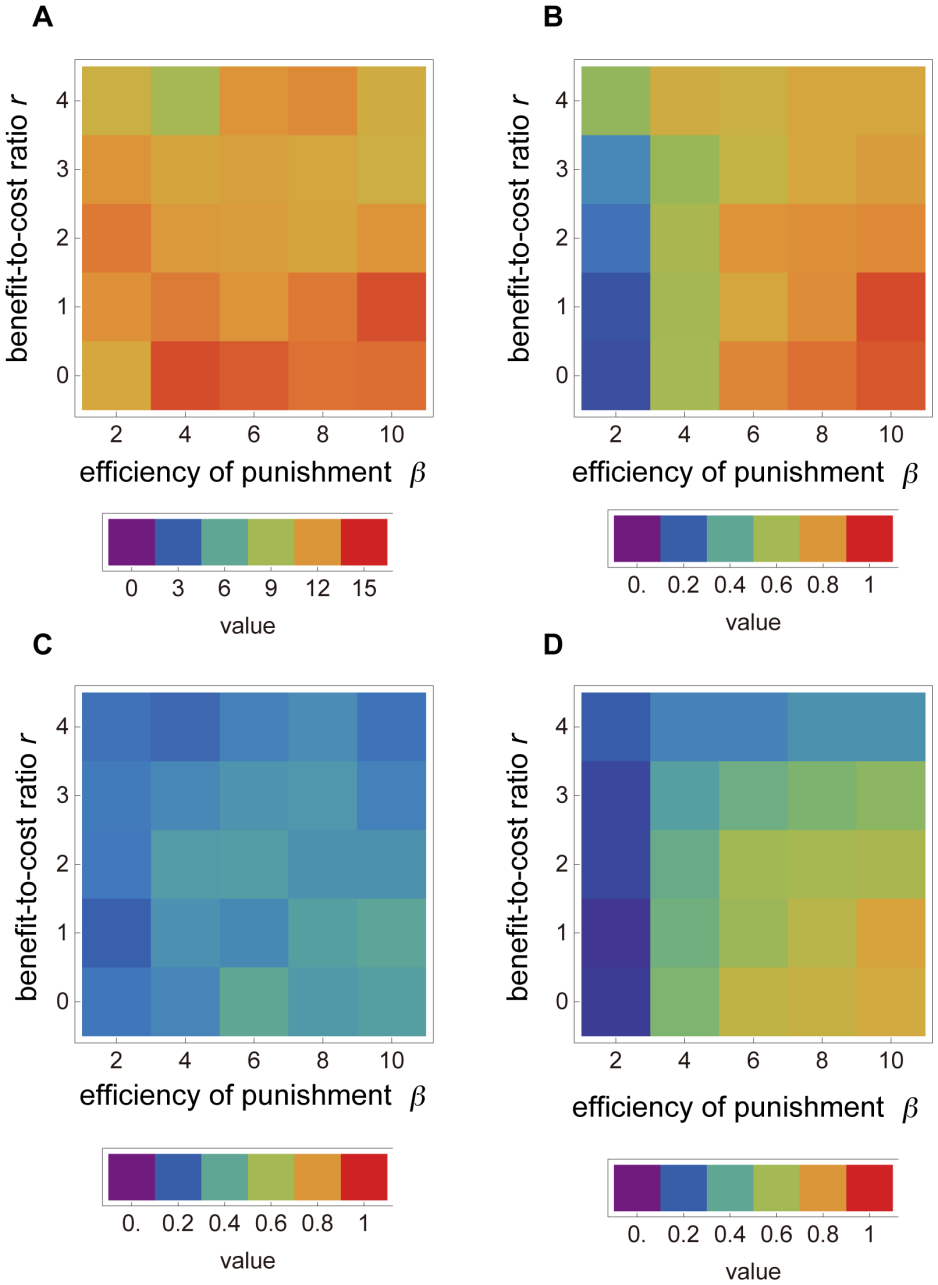
Effects of parameters on evolution in the spatially structured condition. The horizontal and vertical axes represent the effectiveness of public goods (*r*) and punishment (*β*), respectively. Each data block is the average of 50 trials. (A), (B), (C), and (D) represent the average values of *a* (strictness), *x* (cooperation level), *f* (punishment level), and *u* (punishment threshold) at 1,000,000 generations, respectively. Deeper color means that the trait evolved to a higher value. Each bar below each graph presents the relationship between a value and color.

**Figure 6:**
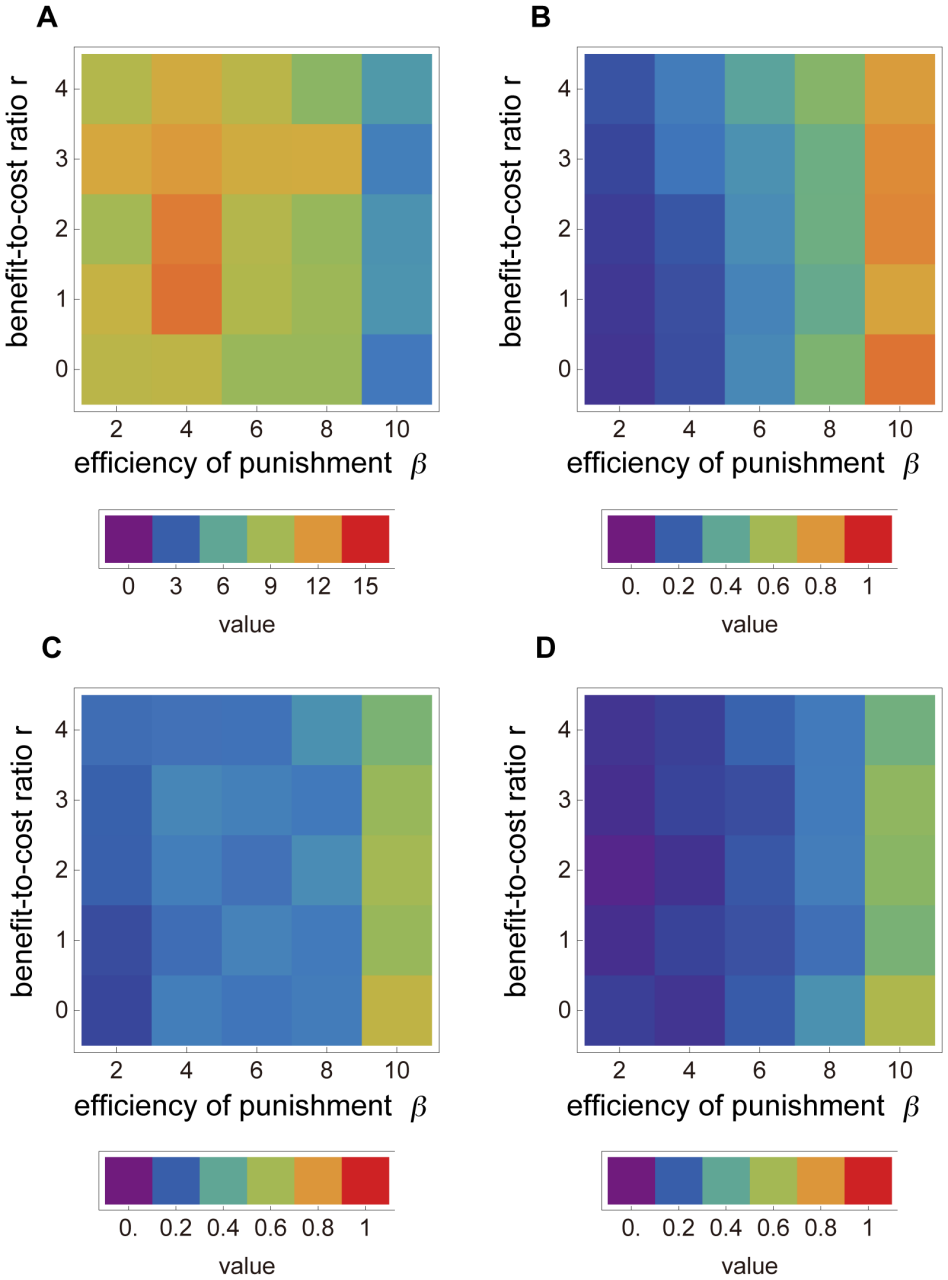
Effects of parameters on evolution in the random-matching condition. The horizontal and vertical axes represent the effectiveness of public goods (*r*) and punishment (*β*). (A), (B), (C), and (D) represent the average values of *a* (strictness), *x* (cooperation level), *f* (punishment level), and *u* (punishment threshold) at 1,000,000 generations, respectively. Deeper color means the trait evolved to a higher value. Each data block is the average of 50 trials. Each bar below each graph presents the relationship between a value and color.

We also examined the outcome when strictness (*a*) was not fixed but assumed to be an adaptive trait. We analyzed how two parameters, *r* and *β* (the effectiveness of the public goods and that of the punishment, respectively), affected the evolution of each trait in the spatially structured and random-matching conditions, respectively (Figs. 5 and 6). In the random-matching condition, the value of *β* had to be sufficiently high and higher than that in the spatially structured condition for the coevolution of punishment and cooperation. Strictness (*a*) was higher with a higher cooperation level in the spatially structured conditions (Fig. 5) but lower with a higher cooperation level in the random-matching condition (Fig. 6). By contrast, higher levels of cooperation and punishment occurred in both conditions when the scale factor of public goods, *r*, was smaller.

The efficiency of punishment *β* had to be sufficiently high for cooperation to increase in the spatially structured and random-matching conditions because the indirect advantage of fitness of punishment must outweigh the direct cost of punishment. This result is common in both modeling and experimental studies [20].

In contrast, the efficiency of the public goods *r* had to be sufficiently low in the spatially structured condition. Cooperation has two negative effects on the evolution of cooperation in the viability model: (i) it is costly and (ii) it reduces opponents’ death probabilities because the focal individual gives a benefit, the function of *r*, to opponents and then helps to increase the opponent’s score in the viability model. A higher *r* facilitates the second negative effect but restrains the first effect. As a result, the evolved cooperation level is not high in the high *r* condition in both Fig. 5 and 6. This result is counterintuitive from the viewpoint of reality. If the viability model can be introduced into the experiment, we can compare our results in the spatially structured condition with the experiment and discuss whether the viability model actually has a low cooperation level with higher *r*.

It may seem strange that strictness evolves at a higher level in the random-matching condition when *β* is low (Fig. 6A), but this is a trivial result. When this parameter is low in the random-matching condition, neither cooperation nor punishment evolves (Fig. 6B–D), and so punishment is only a cost. Strict punishment can avoid the cost of punishment more than graduated punishment can, even when the severity of punishment is low; an individual is never punished if his/her propensity of altruism (*x*) is higher than the threshold in strict punishment, whereas an individual is punished even though his/her *x* is higher than the threshold in graduated punishment.

### The Mathematical Model of the Random-matching Condition

To determine whether graduated punishment can evolve in the random-matching condition, we devised a mathematical equation to describe the random-matching condition. The assumption in the evolutionary simulations is so complicated that we simply reassume that a focal individual and *z* opponents are randomly chosen from the entire population. Then *z* +1 members play a public goods game and the score of the focal player in the game gives the death rate (eq. 5). If *z* = 8 in this model is considered as eight opponents in the random-matching condition, this analytical result would predict the evolutionary simulation outcomes. To investigate the evolution of continuous traits, the cooperation level *x*, severity of punishment *f,* and threshold of punishment *u*, we can obtain the invasion fitness or the mutant’s growth rate following adaptive dynamics [47], [48]. Adaptive dynamics is a mathematical framework that deals with eco-evolutionary problems based on simple assumptions such as rare mutations or small mutational effects [49]. For simplicity, we assume that strictness of punishment *a* is a constant.

**Figure 7:**
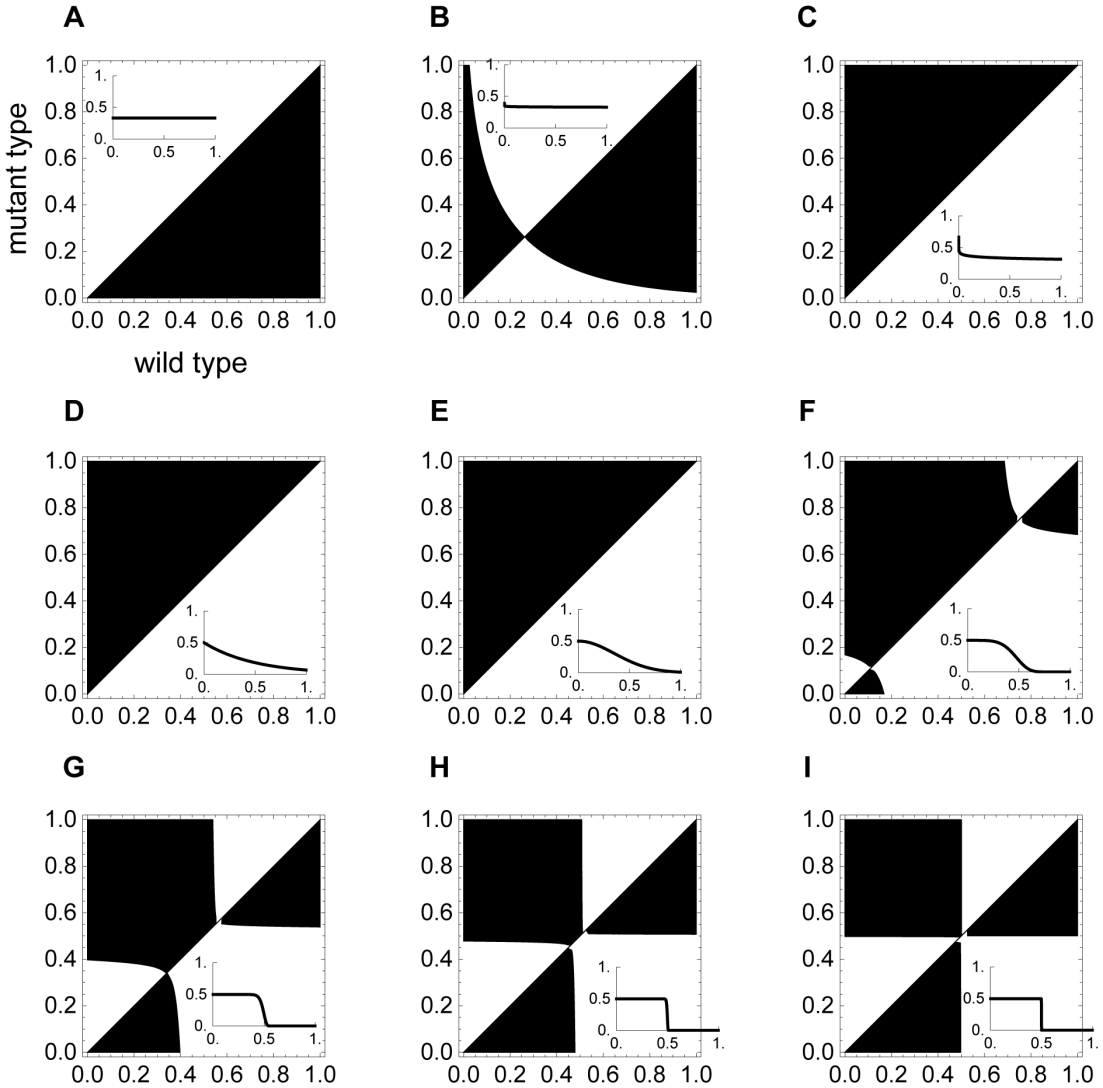
Pairwise invasibility plots (PIPs) of cooperation level when other traits are not adaptive. The horizontal and vertical axes represent the wild and mutant type, respectively. The black region means the mutant type can invade the population occupied by the wild type (*I* >0 in Eq. 6), and the white region means the mutant type cannot invade (*I* <0). The black diagonal means *I* = 0. The small graph in each figure presents the punishment cost assumed in each of (A)–(I). In each small graph, the horizontal and vertical axes represent the opponent’s cooperation level and the punishment cost of the focal player. Parameters are *z* = 8, *r* = 3, *β* = 10, *d* = 0.1, and *u* = 0.5; (A) *a* = 0.0 and *f* = 0.9; (B) *a* = 0.01 and *f* = 0.9; (C) *a* = 0.07 and *f* = 0.9; (D) *a* = 1 and *f* = 0.5; (E) *a* = 2 and *f* = 0.9; (F) *a* = 5 and *f* = 0.9; (G) *a* = 20 and *f* = 0.9; (H) *a* = 100 and *f* = 0.9; and (I) *a* = 1,000 and *f* = 0.9.

Eq. A2a is the fitness gradient of *x* when other traits are fixed. Fig. 7 (*r* = 3, *β* = 10, *z* = 8) are the pairwise invasibility plots of *x* when values close to the evolved values of *f* and *u* in Fig. 3B are substituted into *f* and *u* in Eq. A2a. Fig. 7 shows that when the punishment level remains the same regardless of the opponent’s cooperation level (*a* = 0), the population is eventually dominated by defectors (*x* = 0). When *a* = 0.01, the value of *x* converges to a low level, which is an evolutionary stable strategy (ESS). When punishment becomes graduated (*a* is 0.07, 1, and 2 in Fig. 7), the cooperation level converges to 1 (Fig. 7C–E). When punishment becomes strict, there are at least two singular points in Fig. 7F–I, and these figures imply that the cooperation level may converge to the higher value of the singular point when *x* starts from a high value. The stricter the punishment, the lower the converged value of *x*. Even though *f* and *u* are not evolutionary traits in Fig. 7, these theoretical results can roughly predict the simulation outcomes of *x* in Fig. 3B.

For *x*, *f,* and *u* as independent evolutionary traits, the invasion gradients obtained are presented in Appendix S1. Eq. A2b and A2c indicate that traits *f* and *u* always increase through the evolutionary process when *β*>*z*. This result predicts the simulation outcomes of *f* and *u* in the random-matching condition (Fig. 6C–D).

## Discussion and Conclusion

The major finding of this study is that strict punishment is more effective in increasing cooperation in a spatially structured population and graduated punishment is more effective without spatial structure. In the previous section, we have discussed the effects of parameters such as *r* and *β* on the evolution of cooperation and punishment. With a spatial structure, as Nakamaru and Dieckmann noted, stricter punishment is more effective because the punisher can target less cooperative neighbors [37]. If punishment is strict (e.g., Fig. 1B), the cooperation level of individual *i* (*x_i_*) should be higher than a neighbor’s threshold of punishment (*u_j_*), if the individual is to avoid punishment (Fig. 3A). Otherwise, the individual is punished at the highest level. If an individual has an increased threshold of punishment (*u_j_*), neighbors immediately increase their cooperation levels. Thus, the cooperation level and the punishment threshold evolve together within neighborhoods, and the evolution of cooperation and punishment levels increase when the punishment is more severe.

However, the results are different when players interact with randomly chosen others. First, previous studies have proven that the viability model can promote the evolution of cooperation and punishment in a completely mixed population [23], [30]. The present study demonstrates why graduated punishment rather than strict punishment is more effective in the completely mixed population when the viability model is assumed. Opponents in games are randomly chosen from the population in each turn. Thus, the opponents’ thresholds are not always lower than the cooperation level of a focal player, and a strict punisher (focal player) must pay the maximum cost in vain to punish opponents; further, the same opponents may not play the game with this focal player again. However, graduated punishment is a good way to reduce the punisher’s cost when individuals interact with randomly chosen others. In every game stage, each individual plays a game with various individuals with different cooperation levels. Graduated punishers do not give much damage to an individual with a higher cooperation level. Also, a lower cooperation level reduces the damage from graduated punishment in comparison with strict punishment, regardless of the next opponent’s cooperation level and threshold of punishment level. Thus, graduated punishment can increase cooperation without spatial structure.

From an evolutionary psychology viewpoint, if a function of punishment becomes experimentally clear, we can speculate the type of situation that would cause the evolution of punishment behavior in humans. Strict punishment would evolve in a small, fixed society, such as a band, whereas graduated punishment would more likely evolve in a fluid society.

Another possible hypothesis is that people change their punishing function depending on the relationship with their opponents. Experimental studies have shown that membership of the opponent in the in-group or the out-group determines the punishment strategy [50]–[52]. Based on our study, we can infer that the strategy will be adaptive if people employ strict punishment to in-group members and graduated punishment to out-group members. This is because members of the same group often encounter each other, which is similar to the spatially structured condition. Individuals will encounter others in different groups only occasionally, which corresponds to the random-matching condition.

These hypotheses should be tested in psychological experiments. Although people have a tendency to punish defectors, the function of this type of punishment has not, to our knowledge, been discussed. The experiments of Egas and Riedl have some relevance to our model in that they mention the importance of a punishment threshold for maintenance of cooperative behaviors [20]. Although strictness of punishment was not a factor, and they used linear regressions to represent punishment increases in relation to cooperation decreases, their experimental settings would be useful in studies of people’s punishment functions with appropriate modifications.

Experimental studies have shown the existence of anti-social punishers [50], [53] and the theoretical studies show that anti-social punishment collapses the evolution of cooperation and punishment [54]–[56]. However, it is highly possible that the social or innate motivation to punish cooperators is completely different from the motivation to punish defectors from the viewpoint of reality and social context. For example, envy or malicious intent might make people punish cooperators, while the anger provoked by social justice might make people punish defectors. Herrmann et al. [53] demonstrated that the weak norms of civic cooperation and the weakness of the rule of law in a country are predictors of anti-social punishment. This implies that social structure influences punishment behavior. Therefore, anti-social punishers and punishers who punish cooperators belong to socially or innately different categories and should not be dealt with together. In future, we will formulate appropriate models that will test assumptions regarding what causes people to punish cooperators in order to investigate the emergence of anti-social punishment in our society.

From this viewpoint, it would be useful to study the coevolution of functional forms of punishment and cooperation without assuming anti-social punishment. In our society, graduated sanctions are used for resource management and in the sanction system used by the police [40], [41]. Peoples’ minds are equipped with graduated punishments, so that they may establish a graduated sanction system for resource management and for sanctioning law violators. If this is true, our study can prove why we have a graduated sanction system in our society.

However, future studies should examine what happens when anti-social punishment is introduced in our current model. The mathematical analysis (see Appendix S1) demonstrated that cooperation and punishment did not evolve when players behave as anti-social punishers (the value *a* is fixed and less than zero). When we assume that the evolutionary trait *a* is between −infinite and +infinite, it is difficult to predict whether a new result would follow the previous theoretical studies [54]–[56] because these studies, on the evolution of anti-social punishment, assumed discrete strategies, and then mutation changed non-punishers to punishers and cooperators to defectors. However, our model does not exhibit this behavior because we assume that, when mutation occurs, the offspring’s trait is normally distributed around the parental trait with standard deviation. For example, mutation changes non-punishers to players who punish little if the standard deviation is small. Therefore, the difference in the strategy space between the previous theoretical studies [54]–[56] and our model may cause different evolutionary dynamics, which may depend on the initial condition and the magnitude of standard deviation. When the initial population is monomorphic, the initial value of *a* is positive and high, and the standard deviation is small, the result may be the same as that observed in this study. However, when the initial value of *a* is equal to or less than zero and the standard deviation is very high, anti-social punishment may collapse the evolution of cooperation and punishment in both random-matching condition and spatially structured condition. However, when the initial value of *a* is zero and the standard deviation is small, we require further simulations to investigate whether cooperation and punishment evolve.

Numerous studies have examined cooperation and punishment, but the number that have addressed continuous strategies of cooperation and punishment is far from adequate. The strictness of punishment or of any other functional strategy is an important consideration in the punishment’s success, as this study has proven. Our study provides further information for application in various experiments, simulations, and field work. Our approach can be applied to adaptive punishment or reward, which would change the effort of punishment or reward, depending on the success of cooperation [21], [22], whereas many theoretical studies on punishment assume that the cost of punishing and being punished is fixed for simplicity. In future research, we plan to examine specifically the evolution of severely punishing repeat offenders, a subject that applies to our real society in the punishment of criminals and certain long-standing common-pool resource institutions [41], [42], [44].

## Supporting Information

Appendix S1 **Mathematical analysis of the random-matching condition.**
(DOCX)
