# Correction: Strict or Graduated Punishment? Effect of Punishment Strictness on the Evolution of Cooperation in Continuous Public Goods Games

**DOI:** 10.1371/annotation/ac3a8940-0e84-4f2d-a88d-3ecfebccc9fe

**Published:** 2014-01-22

**Authors:** Hajime Shimao, Mayuko Nakamaru

The current title for Figure 3 is incorrect. The correct title should be "Evolution of cooperation level, punishment severity, and punishment threshold when strictness of punishment is fixed."

