# Supplementary material for: Strict or Graduated Punishment? Effect of Punishment Strictness on the Evolution of Cooperation in Continuous Public Goods Games
Source: PLoS One. 2013 Mar 28;8(3):e59894. doi: 10.1371/journal.pone.0059894 (PMC3610843; doi:10.1371/journal.pone.0059894)
Supplement: Appendix S1 — Mathematical analysis of the random-matching condition. (DOCX) [file pone.0059894.s001.docx]

*Supporting information for "Strict or graduated punishment? Effect of punishment strictness on the evolution of cooperation in continuous public goods games," H. Shimao and M. Nakamaru*

Appendix S1

Let $\rho$ and $\rho_{m}$, respectively, be the frequency of the wild type whose traits are *x*, *f*, and *u*, and that of the mutant type whose traits are *x_m_*, *f_m_*, and *u_m_*. The time differential equation of $\rho_{m}$ is obtained as

$\begin{matrix} \frac{d\rho_{m}}{dt}=\rho\sum_{i=0}^{z-1} \rho^{i}\rho_{m}^{z-i}\frac{z!}{i!\left(z-i\right)!}c\exp\left[-ds_{w,i}\right]\frac{z-i}{z} \\ -\rho_{m}\sum_{i=1}^{z} \rho^{i}\rho_{m}^{z-i}\frac{z!}{i!\left(z-i\right)!}c\exp\left[-ds_{m,i}\right]\frac{i}{z} \end{matrix}$ , (A1)

in which *s_w,i_* is the total score of a focal player who adopts the wild type and plays the public goods game with *i* wild types and (*z* − *i*) mutants. Let *s_m,i_* be the total score of a focal player who is a mutant and plays the public goods game with *i* wild types and (*z* − *i*) mutants. The first term in the right side of eq. A1 means the increase of the mutants: the focal individual with the wild type’s traits plays the game with *i* wild types and (*z* − *i*) mutants and then dies with the death probability defined by eq. 5. After the individual’s death, the site previously occupied by the focal individual becomes a new empty site. Next, one mutant is randomly chosen from *z* opponents, who then colonizes the empty site.

The second term means the decrease of the mutants: the focal individual with the mutant type’s traits plays the game with *i* wild types and (*z* − *i*) mutants and then dies with the death probability defined by eq. 5. After the individual’s death, the site previously occupied by the focal individual becomes a new empty site. Next, one wild type is randomly chosen from *z* opponents, who then colonizes the empty site.

It is assumed that the frequency of mutants is rare in the population and $\rho_{m}^{2}$ is assumed to be zero. Therefore, eq. A1 can be simplified as follows:

$\frac{d\rho_{m}}{dt}\approx c\rho^{z}\rho_{m}\left(c\exp\left[-ds_{w,z-1}\right]-c\exp\left[-ds_{m,z}\right]\right)\equiv c\rho^{z}\rho_{m}I$,

where *I* is defined as the invasion fitness or the mutant’s growth rate.

The gradients of each trait are followed as if the traits are independent:

$\begin{matrix} \frac{dx}{dt}\propto\left.\frac{d}{dx_{m}}I\right|_{x_{m}=x,f_{m}=f,u_{m}=u} \\ =d\left[-1+\left(\beta z-1\right)\left(af\left(\frac{x^{a-1}}{u^{a}}\right)\exp(-(\frac{x}{u})^{a})\right)\right]\exp\left(-dG\right) \end{matrix}$ , (A2a)

$\frac{df}{dt}\propto\left.\frac{d}{df_{m}}I\right|_{x_{m}=x,f_{m}=f,u_{m}=u}=\left(\beta-z\right)d\exp\left(-\left(\frac{x}{u}\right)^{a}\right)\exp\left(-dG\right)$ , (A2b)

$\frac{du}{dt}\propto\left.\frac{d}{du_{m}}I\right|_{x_{m}=x,f_{m}=f,u_{m}=u}=\left(\beta-z\right)\frac{adfx^{a}}{u^{a+1}}\exp\left(-\left(\frac{x}{u}\right)^{a}\right)\exp\left(-dG\right)$, (A2c)

in which *G* = $x\left(r-1\right)-\left(1+\beta\right)zf\exp\left(-\left(x/u\right)^{a}\right)$.

Eq. A2b and A2c imply that if $\beta$ > *z*, the values of *f* and *u* increase with evolutionary time. Other parameters do not affect the sign of eq. A2b and A2c if *u* is not zero in eq. A2c. If *z* = 8 in this model is considered as eight opponents in the random-matching condition, this analytical result can predict the evolutionary simulation outcomes in Fig. 7.

In *a* = 0, the sign of eq. A2a is always negative. This indicates that the cooperation level converges to zero (only for defectors) if the punishment level is constant, regardless of the opponent’s cooperation level. If *a* is less than zero, in which case cooperators are punished, the sign of eq. A2a is negative. If *a* is higher than zero, the sign of eq. A2a is dependent on other parameters.
